# Tracking Proactive Interference in Visual Memory

**DOI:** 10.3389/fpsyg.2022.896866

**Published:** 2022-05-18

**Authors:** Tom Mercer, Ruby-Jane Jarvis, Rebekah Lawton, Frankie Walters

**Affiliations:** Centre for Psychological Research, School of Psychology, University of Wolverhampton, Wolverhampton, United Kingdom

**Keywords:** proactive interference, visual memory, time, decay, interference, recent probes task

## Abstract

The current contents of visual working memory can be disrupted by previously formed memories. This phenomenon is known as proactive interference, and it can be used to index the availability of old memories. However, there is uncertainty about the robustness and lifetime of proactive interference, which raises important questions about the role of temporal factors in forgetting. The present study assessed different factors that were expected to influence the persistence of proactive interference over an inter-trial interval in the visual recent probes task. In three experiments, participants encoded arrays of targets and then determined whether a single probe matched one of those targets. On some trials, the probe matched an item from the previous trial (a “recent negative”), whereas on other trials the probe matched a more distant item (a “non-recent negative”). Prior studies have found that recent negative probes can increase errors and slow response times in comparison to non-recent negative probes, and this offered a behavioral measure of proactive interference. In Experiment 1, factors of array size (the number of targets to be encoded) and inter-trial interval (300 ms vs. 8 s) were manipulated in the recent probes task. There was a reduction in proactive interference when a longer delay separated trials on one measure, but only when participants encoded two targets. When working memory capacity was strained by increasing the array size to four targets, proactive interference became stronger after the long delay. In Experiment 2, the inter-trial interval length was again manipulated, along with stimulus novelty (the number of stimuli used in the experiment). Proactive interference was modestly stronger when a smaller number of stimuli were used throughout the experiment, but proactive interference was minimally affected by the inter-trial interval. These findings are problematic for temporal models of forgetting, but Experiment 3 showed that proactive interference also resisted disruption produced by a secondary task presented within the inter-trial interval. Proactive interference was constantly present and generally resilient to the different manipulations. The combined data suggest a relatively durable, passive representation that can disrupt current working memory under a variety of different circumstances.

## Introduction

Proactive interference (PI) occurs when previously established memories disrupt the retention of newer information (Ries et al., 2021). While the ability to manage PI has been considered a key function of working memory (Engle, 2002), the detrimental effects of PI on working memory are well documented (see Jonides and Nee, 2006). PI is a key process in leading models of memory (e.g., the SIMPLE theory proposed by Brown et al., 2007), but it is also a useful process for those studying forgetting as PI’s presence indicates the continued availability of an old memory. PI’s ability to indicate the persistence of an unnecessary, residual representation is particularly relevant for temporal models of forgetting. For example, theories relying on a decay process (e.g., Barrouillet et al., 2004) expect memories to fade as a function of the absolute amount of time that has passed since encoding, and so from this view PI should be time limited. Similarly, temporal distinctiveness theories (e.g., Brown et al., 2007) expect memories to become harder to retrieve according to the relative amount of time separating different events in memory, meaning PI should decline following longer delays.

The lifetime of PI was explored by Berman et al. (2009), who examined the role of decay in verbal short-term memory. To measure PI, the recent probes task was used, which is a modified variant of the Sternberg task (see Monsell, 1978). Participants were asked to remember four targets over a short delay, followed by a single probe. The task was to determine whether the probe matched any of the current targets, and on Positive trials there was a match between one of the targets and the probe. Of more interest were cases when there was a mismatch and on the crucial recent negative (RN) trials, the probe matched a target from the *previous* trial. These RN trials were then compared against non-recent negative (NRN) trials, where the probe was novel. Performance was consistently slower on RN compared to NRN trials, which indicated PI was present. Berman et al. (2009) also varied the inter-trial interval separating one trial from another, meaning that at longer inter-trial intervals, there was plenty of time for memories of events from the immediate past to decay. Yet this manipulation seemed to have no impact on PI, which remained present at the longest delays tested. Given that there was no need to actively maintain targets from a previous trial once that trial had ended, these findings appear to indicate the presence of a passive, residual memory that persists over the inter-trial intervals examined.

Campoy (2012) did adapt the recent probes task to introduce shorter inter-trial intervals, and here there was a reduction in PI after the shortest delays. However, this was confined to a very limited time window – the PI effect was strongest for inter-trial intervals lasting just 600 ms but diminished at the subsequent inter-trial intervals. Campoy suggested that there may be an initial, very rapid decay, but the PI effect did continue to persist at subsequent delays (albeit less strongly).

One possible explanation for persistent PI in verbal memory concerns the stimuli employed – familiar words (Berman et al., 2009) or numbers (Campoy, 2012). If these verbal stimuli were replaced with non-verbal stimuli, a reduction in PI could reasonably be expected. While PI has been reported for an array of visual stimuli (e.g., Postle et al., 2004; Makovski and Jiang, 2008; Cyr et al., 2017), research has also shown that unfamiliar visual material undergoes rapid forgetting. In several studies asking participants to actively retain such stimuli over brief delays, a drop in performance has been recorded as the interval separating encoding from retrieval is lengthened (e.g., Ricker and Cowan, 2010, 2014, Pertzov et al., 2013; Mercer and Barker, 2020). A similar effect, or an even more pronounced effect, should be expected for the passively maintained visual memories underlying PI.

Surprisingly, however, McKeown et al. (2014) found that PI for such visual stimuli was robust over the passage of time. They adapted the recent probes task for use with abstract visual patterns, varying both the retention interval separating the targets from the probe, and the inter-trial interval separating one trial from another. In two experiments, a PI effect was found, as manifested in slower and less accurate responding to RN probes, compared to NRN probes. This effect was not influenced by the inter-trial interval, though there was evidence that task performance worsened at the longer retention interval.

To account for these findings, McKeown et al. (2014) proposed a dual-process active-passive model. According to this theory, actively retaining visual memories causes their degradation, due to faulty attentional maintenance. Conversely, the passively maintained visual memories behind PI are not subjected to this degradation and persist. As such, this model predicts PI to be invariant to the passage of time.

However, it is unclear whether McKeown et al.’s (2014) temporally robust PI persists under all circumstances, as their participants encoded a relatively small number of unique stimuli on each trial. Under different experimental manipulations, PI may be more susceptible to the inter-trial interval and there have been some demonstrations of time-sensitive PI. In another recent probes task, Mercer and Duffy (2015) had participants encode three complex, unfamiliar visual stimuli on each trial. PI was documented on both response time and accuracy measures at a short 300 ms inter-trial interval, but this disappeared after an 8.3 s delay. More recently, McKeown et al. (2020) conducted five experiments with visual material in the recent probes task. In two of these experiments, there was some evidence for PI declining over the inter-trial interval, though not in the other three.

In a different PI procedure, Shoval and Makovski (2021) presented four images for encoding, followed by a retention interval lasting 500 ms or 4 s. A probe was then displayed. In a PI-prone condition, the same set of stimuli were used throughout the procedure, heightening PI, whereas in a PI-free condition, stimuli were unique on each trial. Results showed reduced PI following a longer retention interval, but this was non-significant and there is uncertainty about the wider robustness of PI. Demonstrations of time-insensitive PI (e.g., McKeown et al., 2014) relate to other findings showing that PI in visual memory can be durable, such as Hartshorne’s (2008) discovery of PI persisting over several trials. Conversely, reports of time-sensitive PI (e.g., Mercer and Duffy, 2015) are compatible with studies finding transient or limited PI (e.g., Makovski and Jiang, 2008; Oberauer et al., 2017), and evidence that PI does not influence working memory capacity (e.g., Lin and Luck, 2012; Balaban et al., 2019).

In summary, there is great uncertainty about the temporal persistence of PI for visual stimuli, and some contradictory findings. This is problematic, as the survival or loss of PI is relevant to understanding forgetting, and particularly the role of time in memory loss. Given that PI sometimes appears to be sensitive to time intervals, it is possible that other factors influence the reported effect, and this was investigated in the present study. This study had three major aims. First, it aimed to assess the overall impact of PI in a recent probes task using unfamiliar visual stimuli. This is necessary given prior contradictory findings concerning PI. Second, the study aimed to determine whether factors that were not controlled or tested in prior studies influenced the lifetime of PI. While several studies have examined the influence of the inter-trial interval on PI in visual memory (e.g., McKeown et al., 2014, 2020; Mercer and Duffy, 2015), there has been little attempt to investigate the factors that may affect the temporal persistence of PI. To do this in the current study, the reported experiments assessed whether the effect of PI over short and long inter-trial intervals was affected by the number of targets to be remembered (Experiment 1) or the number of stimuli forming the target set (Experiment 2). Third, the study examined whether PI could be eliminated by a secondary task presented within the inter-trial interval, as may be expected if interference processes have greater responsibility for forgetting than temporal processes. This was tested in Experiment 3.

## Experiment 1

The first study investigated the role of array size in the persistence of PI. To assess the persistence of PI, two inter-trial interval lengths were used –short (300 ms) and long (8 s) – matching two of the delays used by Mercer and Duffy (2015). These interval lengths were chosen as that previous study did find a temporal recovery from PI, showing that the difference between the short and long delays was sufficient. In addition, the shortest inter-trial interval was briefer than those used in some other similar studies (McKeown et al., 2014, 2020), which may increase the chances of detecting changes to PI.

The array size manipulation concerned the number of targets that had to be memorized, which was either small (two targets) or large (four targets). Visual working memory has a well-known capacity limitation (see Luck and Vogel, 2013), and therefore task performance was expected to be much lower with a large array size. Of most relevance, however, was the interaction between array size and PI. It is possible that PI rapidly fades following longer delays with bigger array sizes, and in prior work time-sensitive PI was documented when three targets were encoded on each trial (Mercer and Duffy, 2015), but not when only two targets were presented (McKeown et al., 2014).

**FIGURE 1:**
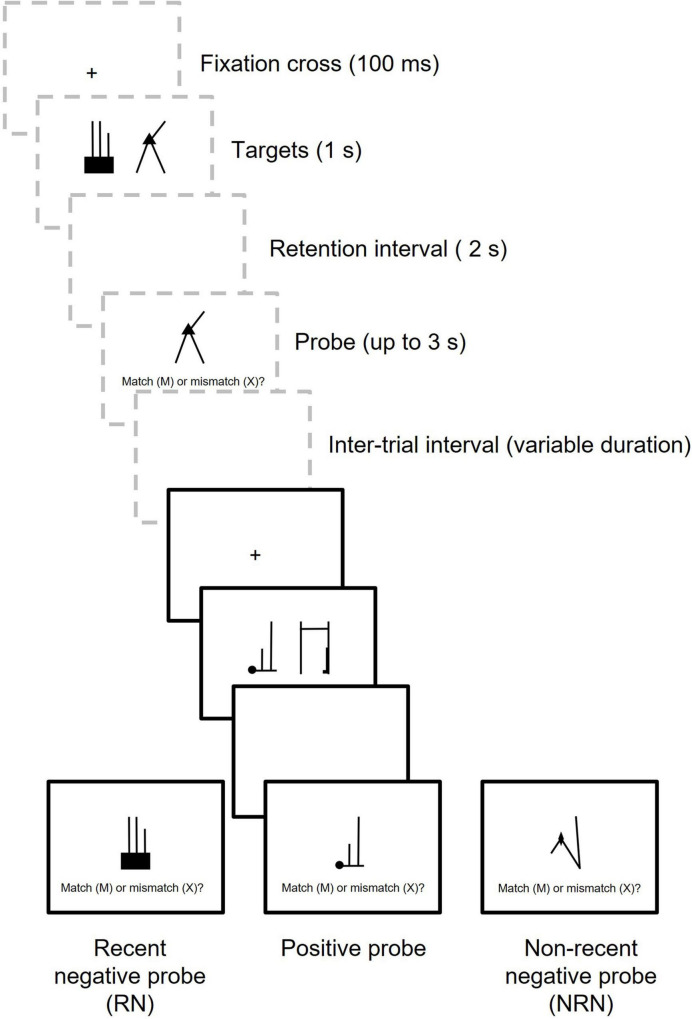
Diagram showing two trials in the recent probes task. Trial *N*-1 is shown in dashed gray boxes and Trial *N* is shown in black boxes. In this example, two targets are encoded on each trial.

The present study used the recent probes task to investigate these issues (see Figure 1 for a diagram of the procedure). RN probes were an untested target from the preceding trial (trial *N*-1), whereas NRN probes were untested targets from a more distant trial. Manipulating NRN probes in this way is preferable to using entirely novel NRN probes, as these NRN stimuli have previously been experienced and therefore have an opportunity for encoding – the same as RN stimuli. Fully novel NRN stimuli may be responded to based on their complete unfamiliarity, which could exaggerate the magnitude of any PI effect, so using existing stimuli as NRN probes provides a better comparison for the RN condition.

In terms of the interaction between array size and PI, it is plausible that PI would fade more quickly over the inter-trial interval when four targets were presented, due to the increased load placed on working memory. Retaining items from both the current and previous trial should be more challenging in the large array size condition, leading to the more rapid loss of items encountered in the recent past. This possibility was tested on both accuracy (proportion of correct responses) and response time measures.

### Materials and Methods

#### Participants

Data were collected during the coronavirus pandemic and therefore the experiment was conducted online. The study was advertised on social media and the website ‘‘Psychological Research on the Net,’’^1^ as well as through an internal participant pool and social media. Participants had to consent to the study and confirm they were willing to submit their responses for analysis at the end of the procedure. The experiment was approved by a Faculty Ethics Committee.

The reduction in PI over an inter-trial interval, which was the main effect of interest, has not always been reported in past research. When present, the effect size (η_p_^2^) has varied from 0.16 to 0.23. Taking the smaller effect, an a-priori power analysis (*p* = 0.05, power = 80%) suggested a sample of 46 individuals in a fully repeated measures design. However, effort was made to recruit 92 individuals overall, to account for the addition of a between-groups variable (array size), and to ensure the experiment was suitably powered.

In total, 104 participants finished the procedure, but two individuals asked to withdraw their data and a further four were removed due to numerous missing responses (> 15% of trials had no response). The final sample included 98 individuals (67 females, 28 males, and three non-binary/third gender) aged between 18 and 51 (*M* = 23.90; SD = 7.07; one missing). Forty-eight participants completed the experiment with two targets and 50 had four targets.

#### Materials

Targets and probes were selected from the shape-line (SL) stimuli used in McKeown et al. (2020, Experiments 4–5, see Figure 1 for examples). These are simple stimuli featuring a single shape (a circle, a square, a triangle, a diamond, a star, a cross, an “L” and an “X”) and three lines of different length/orientation. These stimuli were chosen as there are a large number (600), eliminating the need to re-use stimuli across trials, and they have previously been used to demonstrate a PI effect in the recent probes task (McKeown et al., 2020). When the array size included four targets, 64 objects were randomly selected for each of the eight shape types, requiring 512 images in total. Targets were then pseudo-randomly paired together, with the requirement that a specific shape type could not be used more than once within a pair of targets. An additional 32 SL images were used on the practice trials. Arrangements for the two-target array size matched the four-target condition, except 256 SL stimuli were used (including 32 for each shape type), plus a further 18 on practice trials. All images were displayed on a white background and the experiment was designed and run using the Gorilla Experiment Builder (Anwyl-Irvine et al., 2020). Participants could only access the experiment *via* a desktop computer or laptop, and they responded to the probe using a keyboard.

#### Design and Procedure

The experiment used a 2 (array size: two targets vs. four targets) × 2 (probe type: RN vs. NRN) × 2 (inter-trial interval: 300 ms vs. 8 s) mixed design. Probe type and inter-trial interval were within-groups variables, whereas array size was a between-groups variable.

Participants accessed the experiment *via* a web link and were asked to provide their informed consent. As data were collected remotely, several steps were taken to ensure participants understood the task. They were given contact details of the researchers and invited to ask any questions, they had to indicate that they understood the procedure before continuing, the task was explained *via* an instructional video, and they completed eight practice trials prior to the formal procedure. In the main experiment, each trial began with a fixation cross displayed for 100 ms, followed by two or four targets, shown for 1 s. The encoding time remained constant for both the two-target and four-target conditions, in line with other studies of visual working memory capacity (e.g., Luck and Vogel, 1997; Vogel and Machizawa, 2004; Xu et al., 2018).

After an unfilled retention interval lasting 2 s, the probe was displayed for up to 3 s, in the center of the screen. Participants had to press “M” if they believed the probe matched one of the current targets, or “X” if there was a mismatch. Participants had been instructed to respond as quickly as possible, but without sacrificing accuracy. The next trial then began after an inter-trial interval lasting 300 ms or 8 s.

On half of the trials, the probe did match one of the current targets, but there were two types of mismatching trials. On RN trials, the probe matched an untested target from the *previous* trial, whereas probes on NRN trials were an untested target from either trial *N*-5 or *N*-6. Targets could occupy one of either two or four positions, depending on the array size, and probes related to each position an equivalent number of times for each probe type/inter-trial interval duration. The two array size conditions were identical, except for the number of target stimuli used.

The 128 experimental trials were completed in two separate blocks, containing 32 Positive trials, 16 RN trials, and 16 NRN trials. The PI effect has been demonstrated with as few as 12 trials (e.g., Berman et al., 2009; McKeown et al., 2014; McKeown et al., 2020, Experiments 1–3), but this was increased to 16 trials in the present study to maximize the chance of detecting PI, while also following the approach of some previous studies (e.g., McKeown et al., 2020, Experiments 4–5; Mercer and Duffy, 2015).

The order of trials within a block was fixed, due to the requirements of RN and NRN probes, but the block order was counterbalanced. Each trial block contained an equal mixture of inter-trial intervals, and the two blocks were separated by an optional break. In total, participants completed 64 Positive trials, 32 RN trials, and 32 NRN trials, divided equally between the short and long inter-trial intervals. Figure 1 shows two example trials.

### Results

The mean proportion of correct responses in each condition, and mean response times for trials with a correct response only, were calculated for each probe type and inter-trial interval. Trials where participants did not respond within the 3 s time limit were removed, but these were rare.

Given the wide variety of ages, analyses were also performed to check that age was comparable in the two array size conditions. Age distributions were similar (two targets: *M* = 23.29, SD = 6.12; four targets: *M* = 24.49, SD = 7.92), and an independent samples *t*-test did not reveal a significant difference in age between the two conditions, *t*(95) = –0.83, *p* = 0.407, *d* = –0.17. Additionally, age was not correlated with accuracy on the task, *r*(95) = –0.04, *p* = 0.677, though older adults were slower to respond overall, *r*(95) = 0.24, *p* = 0.020.

Data on the Positive trials are uninformative about PI (see Berman et al., 2009) but can provide other insights into task performance. A paired-samples *t*-test revealed that the proportion of correct responses was significantly higher at the longer inter-trial interval (*M* = 0.70), compared to the shorter one (*M* = 0.67), *t*(97) = –2.74, *p* = 0.007, *d* = –0.18. However, an equivalent *t*-test showed response times were slower at the longer inter-trial interval (*M* = 1,052.94 ms), compared to the short one (*M* = 996.32 ms), *t*(97) = –5.14, *p* < 0.001, *d* = –0.25.

#### Proactive Interference Effect for Proportion Correct

**FIGURE 2:**
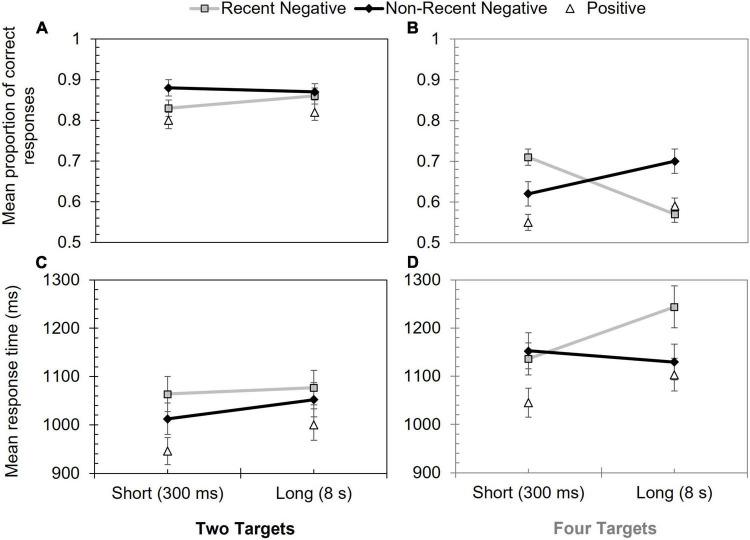
Performance in Experiment 1 according to probe type, inter-trial interval, and array size. Panels **(A,B)** show mean proportion of correct responses for two and four targets, respectively. Panels **(C,D)** show mean response time (ms) for two and four targets, respectively. Error bars show +/–1 SE.

The proportion of correct responses on mismatching trials are shown in Figure 2. A 2 (array size: two targets vs. four targets) × 2 (probe type: RN vs. NRN) × 2 (inter-trial interval: 300 ms vs. 8 s) mixed ANOVA was then used to assess these data. There was a significant effect of probe type, *F*(1, 96) = 7.23, *p* = 0.008, η_p_^2^ = 0.07, with poorer performance on RN (*M* = 0.74) than NRN (*M* = 0.77) trials. Array size also significantly influenced performance, *F*(1, 96) = 60.62, *p* < 0.001, η_p_^2^ = 0.39, as accuracy was lower when four targets had to be remembered (*M* = 0.65), rather than two (*M* = 0.86).

The effect of inter-trial interval, *F*(1, 96) = 1.15, *p* = 0.287, η_*rm p*_^2^ = 0.01, was non-significant, as were the interactions between probe type and array size, *F*(1, 96) = 0.05, *p* = 0.833, η_p_^2^ < 0.01, and inter-trial interval and array size, *F*(1, 96) = 3.87, *p* = 0.052, η_p_^2^ = 0.04 (the latter interaction was close to the significance threshold, but was captured in the three-way interaction, as outlined below).

There was a significant interaction between probe type and inter-trial interval, *F*(1, 96) = 14.99, *p* < 0.001, η_p_^2^ = 0.14, as well as a significant three-way interaction, *F*(1, 96) = 42.34, *p* < 0.001, η_p_^2^ = 0.31. Only the latter interaction was followed-up, however, as the three-way interaction subsumed the two-way interaction.

To break down the higher-order interaction, a PI score was calculated for each inter-trial interval. To factor in the baseline difference between array sizes (lower performance with four targets), the PI score was based on a relative proportion (see also Shoval and Makovski, 2021). RN performance was subtracted from NRN performance, and the resulting score was divided by the RN score. These data can be viewed in Table 1. Positive scores indicated the presence of PI (a lower score on RN than NRN trials). A series of Holm-Šidàk corrected *t*-tests were then used to make specific comparisons between the four scores. At the short inter-trial interval, PI was stronger for two, rather than four, targets, *t*(96) = 3.89, *p* < 0.001, *d* = 0.79. At the longer inter-trial interval this effect was reversed, with stronger PI for the four-target array, *t*(55.5) = –3.25, *p* = 0.004, *d* = –0.65 (values corrected as equal variances could not be assumed). Paired-samples *t*-tests also showed that PI increased in the four-target array as the inter-trial interval was lengthened, *t*(49) = –4.11, *p* < 0.001, *d* = –0.85, whereas in the two-target condition, PI declined at the longer inter-trial interval, *t*(47) = 2.03, *p* = 0.048, *d* = 0.43.

**TABLE 1:** Mean relative PI effect (and SD) for proportion correct and response time in Experiment 1 according to inter-trial interval and array size.

	Proportion correct		Response time	
		
Array size	300 ms	8 s	300 ms	8 s
Two	0.10 (0.19)	0.02 (0.17)	0.05 (0.11)	0.04 (0.16)
Four	–0.10 (0.30)	0.34 (0.69)	0.002 (0.15)	0.11 (0.20)

To complement the frequentist ANOVA, specific Bayesian comparisons were conducted. The Bayes factor (BF^10^) can indicate support for the alternative compared to the null hypothesis. As a guide, BF^10^ values of 3, 10, 30, and 100 can be used to denote moderate, strong, very strong, and extreme support for the alternative hypothesis, respectively (Wagenmakers et al., 2018). Conversely, BF^10^ values below 0.33 offer support for the null hypothesis, with values in the intermediate range (0.33–3) being considered insensitive (Wagenmakers et al., 2018).

Different techniques for calculating the Bayes factor have been outlined, but a particularly useful approach for assessing specific predictions has been provided by Dienes (2014, 2019). This approach is based on a half-normal distribution and requires an expectation of the predicted effect. Here, the estimated effect was based on six experiments reported by Mercer and Duffy (2015) and McKeown et al. (2020). Across these experiments, the average performance difference between RN and NRN probes was 0.06. Using this as the estimated effect size for the present data revealed strong evidence for the alternative hypothesis, supporting the presence of PI (BF^10^ = 26.17). To assess the specific PI effect according to array size, the estimated effect was also 0.06. If a PI effect of this size was present at the short inter-trial interval, but vanished at the long inter-trial interval, the resulting PI effect would be equivalent to 0.06, based on estimations from past work (i.e., the PI effect at short inter-trial interval minus PI effect at long inter-trial interval). Using this information to test the prediction that PI would decline from the short to long inter-trial interval, there was strong evidence for the alternative hypothesis when two targets were encoded (BF^10^ = 10.44). However, there was support for the null hypothesis when four targets were encoded (BF^10^ = 0.06), as PI became stronger at the longer interval.

#### Proactive Interference Effect for Response Time

Response time data (with errors removed) are shown in Figure 2 and were assessed using another 2 (array size: two targets vs. four targets) × 2 (probe type: RN vs. NRN) × 2 (inter-trial interval: 300 ms vs. 8 s) mixed ANOVA. There was a significant effect of probe type, *F*(1, 96) = 11.90, *p* < 0.001, η_p_^2^ = 0.11, with slower performance on RN (*M* = 1,130.18 ms) than NRN (*M* = 1,086.67 ms) trials. Array size affected responding too, *F*(1, 96) = 5.83, *p* = 0.018, η_p_^2^ = 0.06, with slower responses when four targets had to be remembered (*M* = 1,165.53 ms), rather than two (*M* = 1,051.32 ms). The inter-trial interval effect was also significant, *F*(1, 96) = 7.61, *p* = 0.007, η_p_^2^ = 0.07, with slower responses at the longer (*M* = 1,125.54 ms) than shorter (*M* = 1,091.31 ms) delay.

Matching the accuracy analysis, the interactions between probe type and array size, *F*(1, 96) = 0.18, *p* = 0.672, η_p_^2^ < 0.01, and between inter-trial interval and array size, *F*(1, 96) = 0.42, *p* = 0.520, η_p_^2^ < 0.01, were non-significant. However, there were interactions between probe type and inter-trial interval, *F*(1, 96) = 5.23, *p* = 0.024, η_p_^2^ = 0.05, and between all three variables, *F*(1, 96) = 11.82, *p* < 0.001, η_p_^2^ = 0.11.

Following the approach used for accuracy, only the three-way interaction was explored further. A relative PI score was calculated by subtracting NRN response times from the equivalent RN trials and dividing the resulting value by the NRN response time. A positive score indicated PI (slower responding on RN, compared to NRN, probes) see Table 1. A series of Holm-Šidàk corrected *t*-tests were then performed. When comparing the PI effect over the inter-trial interval, it became stronger when four targets were encoded, *t*(49) = –3.07, *p* = 0.012, *d* = –0.63. This effect was not significant at the short-inter-trial interval, nor were other comparisons significant based on the Holm-Šidàk criteria.

Finally, Bayesian tests were used to explore these effects, again based on the approach outlined by Dienes (2014, 2019). The expected PI effect for response times was estimated from data reported by Mercer and Duffy (2015) and McKeown et al. (2020). In the three experiments finding PI on the response time measure, RN probes slowed responding by an average of 36 ms, compared to the NRN probes. Based on this information, a simple PI score was calculated by subtracting NRN response times from the equivalent RN condition. There was extreme evidence for the alternative hypothesis when considering the overall PI effect (BF^10^ = 141.12). When looking at the possibility of PI declining over the inter-trial interval, however, there was insensitive evidence with two targets (BF^10^ = 1.45), giving little differentiation between the null and alternative hypothesis, and support for the null hypothesis with four targets (BF^10^ = 0.23), as the PI effect became stronger after a longer delay.

### Discussion

Experiment 1 investigated whether the persistence of PI was affected by the passage of time and the array size. Responses to RN probes were both less accurate and slower than NRN probes, supporting the presence of PI in visual memory in line with past work (e.g., Hartshorne, 2008; Makovski and Jiang, 2008; McKeown et al., 2014, 2020; Mercer and Duffy, 2015; Cyr et al., 2017; Shoval and Makovski, 2021). As further expected, performance was worse when the array size was larger, following well established capacity restrictions in visual working memory, but the three-way interaction revealed effects in contrast to the main hypothesis. When two targets had to be retained, PI appeared to be time sensitive on the accuracy measure, declining as the inter-trial interval was lengthened from 300 ms to 8 s. While this specific finding was consistent with decay and temporal distinctiveness theories, the equivalent effect was less obvious on the response time measure and PI unexpectedly became stronger at the longer inter-trial interval when four targets had to be remembered. On both performance measures for the large array size, there was an absence of PI at the shortest inter-trial interval, but strong PI at the longest inter-trial interval. This effect cannot be explained by a decay or temporal distinctiveness process; hence the combined set of findings are difficult to reconcile with temporal models of forgetting.

However, given less time was available to encode individual stimuli in the four-target array, it is possible that those targets were less likely to be successfully encoded. The decision to equate encoding time for the two- and four-target sets was influenced by prior research (e.g., Luck and Vogel, 1997; Vogel and Machizawa, 2004; Xu et al., 2018), but the encoding time was lengthy in comparison to prior studies of visual working memory capacity. Furthermore, while incomplete or inadequate encoding could explain performance being lower when four targets were presented, a large reduction in PI may be expected to follow in that condition. That this was not the case shows that PI is affected in unexpected ways when working memory capacity is strained.

## Experiment 2

Where Experiment 1 assessed the effects of array size on the temporal persistence of PI, the second experiment investigated stimulus novelty by manipulating the stimuli pool size. Specifically, the number of stimuli used as targets during the experiment was varied, which relates to two different forms of PI. The traditional recent probes task can index item-specific PI (Postle et al., 2004), which is produced by the direct reoccurrence of an item experienced on an earlier trial. Alternatively, item-non-specific PI can play a role, which builds-up over several trials due to repeated exposure to specific stimuli. For example, Hartshorne (2008) demonstrated a build-up of PI over 10 trials. In Hartshorne’s Experiment 2a, which used a blocked design, participants completed a change detection task with a specific set of stimuli (e.g., colors or shapes) for 10 trials. After the tenth trial, a new set of stimuli were used for the next 10 trials, and so on. Experiment 2b replicated Experiment 2a’s blocked condition, but also added a randomized condition, where the same type of stimuli were not presented in this blocked manner. In Experiment 2a, and the blocked condition of Experiment 2b, performance on the first trial of a block was significantly better than the tenth trial, which may be due to very low PI on the first trial with a new set of stimuli. Further analysis indicated a build-up of PI occurring over 4–5 trials, but no such effect was found in the randomized condition of Experiment 2b.

Additional evidence for the important role of stimulus homogeneity in PI was reported by Endress and Potter (2014; see also Shoval et al., 2020). Endress and Potter (2014) used a rapid serial visual presentation task, where images of everyday objects were shown very briefly. The number of images in the sequence was varied, after which a single probe was presented for response. In the unique condition, all images were novel and shown only once during the encoding sequence, whereas in the repeated condition the same set of images were used across trials. When estimating capacity, it appeared much larger in the unique condition (with minimal PI) compared to the repeated condition (with high PI).

The number of stimuli used to the form the target set may similarly influence the persistence of item-specific PI. For example, Mercer and Duffy (2015) used a set of just nine targets throughout the study, though their combination varied from trial to trial. Conversely, McKeown et al. (2014) used a much larger set of targets, ensuring stimuli were unique from trial to trial. In the former study, PI did decline over the inter-trial interval, but not in the latter study. It is possible that when a small set of targets are employed, both item-non-specific and item-specific PI affect task performance. To manage this, participants may strategically use the passage of time to forget old stimuli. However, this may be less urgent when item-non-specific PI is minimized.

Experiment 2 tested this possibility. Inspired by Endress and Potter (2014), the recent probes task was adopted such that one condition used different stimuli throughout the entire experiment (the unique condition), whereas another condition used a small set of stimuli throughout the experiment (the repeated condition). Stronger PI was expected in the repeated condition, due to the additional presence of item-non-specific PI. This may also create a greater urgency to remove residual information from working memory, which would be manifested in a reduction of PI over the inter-trial interval when stimulus novelty was low.

### Materials and Methods

#### Participants

Data for this experiment were collected online at the same time as Experiment 1. Effort was made to recruit 92 participants, in line with the previous power analysis, but this was challenging. In total, 73 participants finished the procedure, but two asked to withdraw their data and six were removed due to numerous missing responses (> 15% of trials). The final sample included 65 individuals (48 females, 15 males, and 2 non-binary/third gender) aged between 18 and 76 (*M* = 29.50; SD = 12.56, one missing). The final sample size was therefore smaller than desired, but 31 participants were assigned to the repeated condition and 34 were assigned to the unique condition. Participants had to consent to the study and confirm they were willing to submit their responses for analysis at the end of the procedure. The experiment was approved by a Faculty Ethics Committee.

#### Materials

Stimuli and software matched the first experiment, though there were two targets on each trial. To create the repeated condition, eight stimuli were selected and used repeatedly throughout the procedure. Each of the eight items were used as a target on 16 trials and as probes between 12 and 20 times. With the unique condition, each stimulus was used as a target only once and arrangement were very similar to the two-target condition of Experiment 1. NRN probes matched a target from at least four previous trials and the arrangements for NRN trials was equivalent for both stimulus novelty conditions.

#### Design and Procedure

The experiment used a 2 (stimulus novelty condition: unique vs. repeated) × 2 (probe type: RN vs. NRN) × 2 (inter-trial interval: 300 ms vs. 8 s) mixed design. Probe type and inter-trial interval were again within-groups variables, whereas stimulus novelty condition was a between-groups variable. All other procedural arrangements matched the two-target condition of Experiment 1.

### Results

Data coding followed the approach outlined in Experiment 1. As age again varied widely, the distribution in age in the two stimulus novelty conditions was assessed. The mean age in the two conditions was similar (repeated condition: *M* = 30.33, SD = 15.18; unique condition: *M* = 28.76, SD = 9.88), and an independent samples *t*-test did not reveal a significant difference in age between the two groups, *t*(62) = 0.50, *p* = 0.311, *d* = 0.12. Like Experiment 1, age was not correlated with accuracy on the task, *r*(62) = –0.02, *p* = 0.878, though older adults were again slower to respond overall, *r*(62) = 0.32, *p* = 0.010.

Also matching Experiment 1, performance on Positive trials was assessed at each inter-trial interval. A paired-samples *t*-test revealed that the proportion of correct responses was significantly higher at the longer inter-trial interval (*M* = 0.83), compared to the shorter one (*M* = 0.79), *t*(64) = –3.04, *p* = 0.003, *d* = –0.29. An equivalent *t*-test revealed a tendency for slower response times at the longer inter-trial interval (*M* = 998.16 ms), compared to the short one (*M* = 972.11 ms), but this was non-significant, *t*(64) = –1.89, *p* = 0.064, *d* = –0.25.

#### Proactive Interference Effect for Proportion Correct

**FIGURE 3:**
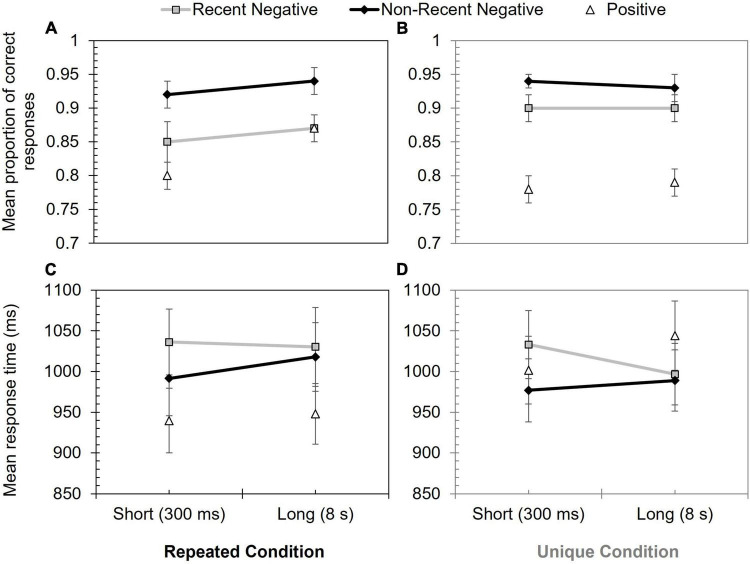
Performance in Experiment 2 according to probe type, inter-trial interval, and stimulus novelty condition. Panels **(A,B)** show mean proportion of correct responses for repeated and unique conditions, respectively. Panels **(C,D)** show mean response time (ms) for repeated and unique conditions, respectively. Error bars show +/–1 SE.

Proportion correct data are shown in Figure 3, and these were explored with a 2 (stimulus novelty condition: unique vs. repeated) × 2 (probe type: RN vs. NRN) × 2 (inter-trial interval: 300 ms vs. 8 s) mixed ANOVA. This revealed a significant effect of probe type, *F*(1, 63) = 29.32, *p* < 0.001, η_p_^2^ = 0.32, due to poorer performance on RN (*M* = 0.88) than NRN (*M* = 0.93) trials. Performance was slightly higher in the unique condition (*M* = 0.92) compared to the repeated condition (*M* = 0.89), but this main effect was non-significant, *F*(1, 63) = 0.85, *p* = 0.359, η_p_^2^ = 0.01.

The interaction between probe type and stimulus novelty condition was marginally significant, *F*(1, 63) = 3.94, *p* = 0.051, η_p_^2^ = 0.06, resulting from a greater PI effect in the repeated (*M* = 0.07) than unique (*M* = 0.03) condition.

All other effects and interactions were non-significant, including the theoretically significant probe type x inter-trial interval interaction: *F*(1, 63) = 0.24, *p* = 0.627, η_p_^2^ < 0.01 [other null effects included inter-trial interval: *F*(1, 63) = 1.26, *p* = 0.267, η_p_^2^ = 0.02; inter-trial interval × stimulus novelty condition: *F*(1, 63) = 1.65, *p* = 0.203, η_p_^2^ = 0.03; stimulus novelty condition × probe type × inter-trial; interval: *F*(1, 63) = 0.35, *p* = 0.554, η_p_^2^ = 0.01].

To supplement this analysis, a PI effect was generated, subtracting RN scores from NRN scores (as performance was relatively similar across conditions, the relative proportion calculated in Experiment 1 was not required). The descriptive statistics are shown in Table 2. Additional Bayesian analyses were then used to assess specific outcomes. Based on the approach used in Experiment 1, comparison of overall RN and NRN performance yielded extreme support for the alternative hypothesis (BF^10^ = 62,934.51) and thereby evidence of PI. When assessing whether this PI declined over the inter-trial interval, there was far less support (repeated condition: BF^10^ = 0.47; unique condition: BF^10^ = 0.78). While these effects were insensitive, they were more compatible with the null hypothesis, and Table 2 indicates the presence of PI at each inter-trial interval. However, as there was a potential interaction between probe type and stimulus novelty condition, a final Bayesian analysis was used to assess the magnitude of PI according to stimulus novelty. This suggested moderate evidence for the alternative hypothesis, with stronger PI in the repeated than unique condition (BF^10^ = 3.72).

**TABLE 2:** Mean PI effect (and SD) for proportion correct and response time in Experiment 1 according to inter-trial interval and stimulus novelty condition.

	Proportion correct		Response time	
		
Stimulus novelty condition	300 ms	8 s	300 ms	8 s
Repeated	0.07 (0.11)	0.07 (0.09)	44.75 (165.45)	12.21 (115.80)
Unique	0.04 (0.10)	0.02 (0.08)	56.16 (98.80)	7.75 (98.73)

#### Proactive Interference Effect for Response Time

Response time data, with errors removed, are shown in Figure 3. Another 2 (stimulus novelty condition: repeated vs. unique) × 2 (probe type: RN vs. NRN) × 2 (inter-trial interval: 300 ms vs. 8 s) mixed ANOVA assessing these data yielded only one effect – a significant effect of probe type, *F*(1, 63) = 11.78, *p* = 0.001, η_p_^2^ = 0.16. This was due to slower performance on RN (*M* = 1,024.07 ms) than NRN (*M* = 993.85 ms) trials.

All other main effects and interactions were non-significant: Probe type × inter-trial interval: *F*(1, 63) = 2.71, *p* = 0.105, η_p_^2^ = 0.04; inter-trial interval: *F*(1, 63) = 0.01, *p* = 0.929, η_p_^2^ < 0.01; stimulus novelty condition: *F*(1, 63) = 0.13, *p* = 0.720, η_p_^2^ < 0.01; inter-trial interval × stimulus novelty condition: *F*(1, 63) = 0.97, *p* = 0.329, η_p_^2^ = 0.02; stimulus novelty condition × probe type × inter-trial interval: *F*(1, 63) = 0.10, *p* = 0.748, η_p_^2^ < 0.01.

Follow-up Bayesian analyses, based on a PI score generated by subtracting NRN response times from RN response times, were then performed. The initial test compared overall response times on RN and NRN trials and supported the presence of PI by revealing substantial support for the alternative hypothesis (BF^10^ = 139.23). When looking at the possibility of time-sensitive PI, comparing the PI effect at the short and long-inter trial intervals, there was anecdotal evidence for the alternative hypothesis (BF^10^ = 2.71), but this was insensitive (see Table 2 for the PI effect data – while a reduction in PI was present, variability was high).

### Discussion

Experiment 2 tested whether the temporal persistence of PI was influenced by stimulus novelty. Replicating Experiment 1, an item-specific PI effect was found for both accuracy and response time. There was also some evidence for item-non-specific PI effect, which was manipulated through the stimulus novelty manipulation (stimuli being repeated or unique). Specifically, the interaction between probe type and stimulus novelty condition was marginally significant, caused by a stronger PI effect in the repeated rather than unique condition, and the Bayesian analysis showed moderate support for the alternative hypothesis. Overall, however, the contribution of item-non-specific PI was modest and there was little evidence for that it affected the persistence of PI over the inter-trial interval. Indeed, compared to Experiment 1, evidence that PI weakened after the 8 s inter-trial interval was limited.

However, the sample size in this experiment was lower than planned (though still sufficient for detecting the within-groups interaction of probe type × inter-trial interval, which required 46 participants based on a η_p_^2^ of 0.16). Assessment of the present effect sizes showed that this interaction was much smaller than originally anticipated – the actual effect sizes for the probe type × inter-trial interval interaction were 0.004 and 0.01, for proportion correct and response time measures, respectively. Based on these actual effects, sample sizes of 1,960 and 782 participants would be required to detect the interaction, indicating that the impact of the inter-trial interval on PI was far smaller than anticipated based on previous studies (e.g., Mercer and Duffy, 2015; McKeown et al., 2020).

## Experiment 3

The first two experiments assessed whether PI declined over the inter-trial interval, and while there was some evidence for this happening on one measure in Experiment 1 (when two targets had to be encoded), an equivalent effect was not reported in Experiment 2. Furthermore, when four targets had to be retained in Experiment 1, PI increased at the longer inter-trial interval.

Instead of being highly sensitive to the passage of time, PI may be more vulnerable to retroactive interference occurring after encoding (e.g., interference produced by an attention-demanding intervening task). Some researchers have argued that such retroactive interference plays a much more substantial role in short-term forgetting than temporal factors (e.g., Farrell et al., 2016), and there is some evidence for this in the recent probes task.

For example, Berman et al. (2009) were able to eliminate the PI effect by inserting another trial between the RN item’s first appearance as a target and its second appearance as a probe (in effect, the RN probe now matched an item from trial *N*-2). While that study used verbal stimuli, Hartshorne (2008) showed that RN probes from more distant trials was weaker than more recent trials in visual memory. In the current data, NRN probes were stimuli shown on a much earlier trial than the present one, and these were responded to more quickly and accurately than the RN probe. Intervening items may therefore be better able to alleviate PI.

To assess the role of (retroactive) interference in the current experiment, a secondary task was introduced into the inter-trial interval on half of experimental trials. While an overall PI effect was predicted, the secondary task may be capable of disrupting memory for an item experienced on trial *N*-1, which would eliminate the difference between RN and NRN trials.

### Materials and Methods

#### Participants

This study used a similar recruitment strategy to the previous experiments, it had received ethical approval and participants provided informed consent prior to beginning the task. As this study used a fully within-groups design, the apriori power analysis suggested a sample size of 46 individuals (with *p* = 0.05 and 80% power). Sixty participants using either a laptop or desktop computer completed the procedure. Three participants were removed due to numerous missing responses and a further four participants asked to withdraw their data. The final sample included 53 individuals (34 females, 15 males, and four not disclosed) aged between 19 and 72 (*M* = 31.43; SD = 13.76; six not disclosed).

#### Materials

Stimuli and software largely matched the previous two experiments, with two targets used on each trial. Additional stimuli were needed to create the distractor condition, and these were selected from Brady et al.’s (2008) database available at https://bradylab.ucsd.edu/publications.html. Sixty-eight distractor images were selected (64 for experimental trials and four for practice trials), with half showing living creatures (e.g., a penguin), and the remainder showing inanimate objects (e.g., a wooden chair).

#### Design and Procedure

The experiment used a 2 (distractor condition: distractor vs. control) × 2 (probe type: RN vs. NRN) repeated measures design. Arrangements for the memory trials resembled the previous studies using two targets, except responses to the probe were undertaken *via* a response button. Underneath the probe were two buttons – one labeled match and one labeled mismatch – and participants had to select their preferred response within 3 s. This was followed by an inter-trial interval lasting 3 s. On distractor trials, a single image was shown in the center of the screen and participants had to decide whether it showed an animate or inanimate object using response buttons presented underneath the images (response buttons were used to make it easier for participants to answer on the memory and distractor trials, without having to use four different keys). The distractor remained on screen for 3 s regardless of when participants responded. In the control condition, the distractor was removed, and the inter-trial interval was an unfilled 3 s gap.

The rationale for the secondary task was based around findings that visual working memory is more sensitive to interruptions (events requiring attention and a response), rather than distraction, involving the passive viewing of an irrelevant item (see Makovski et al., 2006; Clapp et al., 2010; Makovski and Pertzov, 2015). The current secondary task was expected to be attention demanding as it required a response within a limited time window and therefore have the potential to interrupt any maintenance of stimuli seen on the prior trial.

Distractor and control trials were completed in separate blocks containing 32 match, 16 RN and 16 NRN trials. The order of the blocks was counterbalanced, and two different arrangements of the experiment were created. The memory trials used in the distractor condition in Version A of the experiment served as the control condition in Version B, and vice versa. Prior to undertaking the main procedure, participants were given four practice trials without a distractor and a further four practice trials with a distractor. The experiment lasted approximately 30 min.

### Results

Data coding followed the approach outlined in Experiment 1. Participants of various ages completed the task and the correlation between age and task performance was again checked. Matching Experiments 1 and 2, age was not significantly correlated with accuracy on the task, *r*(41) = 0.11, *p* = 0.492, but older adults were slower to respond overall, *r*(41) = 0.46, *p* = 0.002. Finally, paired-samples *t*-tests compared performance in the two distractor conditions (distractor vs. control) on Positive trials, but found no differences for proportion correct, *t*(52) = –0.03, *p* = 0.975, *d* = –0.01, or response time, *t*(52) = –0.79, *p* = 0.432, *d* = –0.13.

#### Proactive Interference Effect for Proportion Correct

**FIGURE 4:**
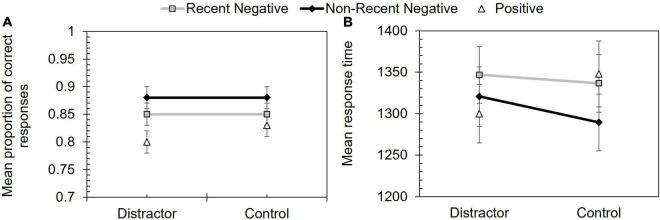
Performance in Experiment 2 according to probe type and distractor condition. Panel **(A)** shows mean proportion of correct responses and panel **(B)** shows mean response time (ms). Error bars depict +/–1 SE.

The proportion correct data are shown in Figure 4 and a 2 (distractor condition: distractor vs. control) × 2 (probe type: RN vs. NRN) repeated measures ANOVA revealed a significant effect of probe type, *F*(1, 52) = 6.34, *p* = 0.015, η_p_^2^ = 0.11. Performance on RN trials (*M* = 0.85) was lower than NRN (*M* = 0.88) trials. However, the effect of distractor condition, *F*(1, 52) = 0.04, *p* = 0.841, η_p_^2^ < 0.01, and the interaction, *F*(1, 52) < 0.01, *p* = 0.975, η_p_^2^ < 0.01, were non-significant.

A Bayesian analysis was then performed, using the same parameters as Experiments 1 and 2. Comparing RN and NRN performance on the PI score (NRN minus RN values; see Table 3) found strong support for the alternative hypothesis (BF^10^ = 26.17). Conversely, when comparing the PI effect in the distractor and control conditions, there was more support for the null hypothesis, though the effect was insensitive (BF^10^ = 0.46).

**TABLE 3:** Mean PI effect (and SD) for proportion correct and response time in Experiment 3 according to distractor condition.

Distractor condition	Proportion correct	Response time
Distractor	0.03 (0.14)	26.13 (179.64)
Control	0.03 (0.11)	47.03 (140.36)

#### Proactive Interference Effect for Response Time

The error-free response time data are shown in Figure 4. Another 2 (distractor condition: distractor vs. control) × 2 (probe type: RN vs. NRN) repeated measures ANOVA showed a significant effect of probe type, *F*(1, 52) = 4.23, *p* = 0.045, η_p_^2^ = 0.08, with slower performance on RN (*M* = 1,341.65 ms) than NRN (*M* = 1,305.07 ms) trials. The effect of distractor condition, *F*(1, 52) = 0.74, *p* = 0.395, η_p_^2^ = 0.01, and the interaction, *F*(1, 52) = 0.63, *p* = 0.432, η_p_^2^ = 0.01, were both non-significant.

A Bayesian analysis, based on same parameters as used in Experiments 1 and 2, found moderate support for the alternative hypothesis when comparing RN against NRN trials (BF^10^ = 4.70). However, comparing the PI effect on response time (RN minus NRN) in the distractor and control conditions yielded an insensitive outcome (BF^10^ = 1.07).

### Discussion

The present experiment assessed whether the addition of a secondary task within the inter-trial interval would disrupt PI. PI was again found on accuracy and response time measures, with less accurate and slower responding for RN than NRN probes. However, there was less evidence that the secondary task affected PI, showing it can resist non-specific interference. PI may therefore be robust to both the passage of time and active disruption from other stimuli.

## General Discussion

The present study investigated the temporal persistence of PI in visual memory. There have been some contradictory findings concerning both the robustness and lifetime of PI in visual memory, with some studies finding minimal (e.g., Oberauer et al., 2017) or transient (e.g., Makovski and Jiang, 2008) PI, which may be sensitive to the passage of time (e.g., Mercer and Duffy, 2015). Other studies have found more enduring PI (e.g., Hartshorne, 2008; McKeown et al., 2014), and the present study intended to determine whether specific factors would affect the lifetime of PI, including array size (Experiment 1), stimulus novelty (Experiment 2), and interference produced by a distracting secondary task (Experiment 3).

Overall, the PI effect was largely robust to these manipulations. In all three experiments, responses to RN probes were less accurate and slower than NRN probes. This discovery of PI supported prior studies (e.g., Hartshorne, 2008; Makovski and Jiang, 2008; Endress and Potter, 2014; McKeown et al., 2014, 2020; Mercer and Duffy, 2015; Cyr et al., 2017; Shoval and Makovski, 2021), but charting PI’s persistence over the inter-trial interval yielded a more complex set of findings. In Experiment 1, PI did decline on the accuracy measure when the inter-trial interval was lengthened, at least when two targets had to be encoded. Additionally, responding on Positive trials was also more accurate at the long rather than short inter-trial intervals in both Experiments 1 and 2, supporting the notion that longer delays between trials is beneficial. These findings support a decay process, where memories of events from the previous trial are forgotten over a delay. They are also consistent with demonstrations of time-dependent forgetting for actively maintained visual information (e.g., Ricker and Cowan, 2010, 2014; Pertzov et al., 2013; Mercer and Barker, 2020). A reduction in PI can be explained by temporal distinctiveness theory too (e.g., Brown et al., 2007), where the distinctiveness of events on the current trial is higher when that trial is temporally isolated from the previous trial, improving recognition accuracy.

However, this effect was more ambiguous on the response time measure in Experiment 1, due to high variability. Experiment 2 also did not find time-sensitive PI with two targets (although the Bayesian analysis showed a possible reduction in PI over the inter-trial interval on the response time measure). Furthermore, when participants had to retain four targets on each trial in Experiment 1, the PI effect *became stronger* at the longer inter-trial interval. This was a highly unexpected finding and the exact reason for its occurrence is unclear, but it does challenge the notion that PI weakens as time since encoding passes. As such, the wider set of findings are problematic for temporal models of forgetting, showing redundant, visual representations can remain available over relatively long delays.

Given that temporal models of forgetting are difficult to reconcile with the full set of data reported here, situations where PI does appear to fade over an inter-trial interval may be better explained by other processes. For instance, in the two-target condition of Experiment 1, which did report time-sensitive PI on the accuracy measure, strategic use of the passage of time to segregate one trial from another may have been responsible for the apparent disappearance of PI. That is, memories of stimuli from trial *N*-1 may not have been forgotten but were instead effectively separated from the current trial and therefore less likely to produce PI (an equivalent effect may have happened on Positive trials).

Importantly, then, a reduction in PI is not necessarily a guarantee of the loss of the underlying memory through a temporal process such as decay, and it may be better conceptualized as the effective management of PI. The difficulty is that it remains unclear as to when the passage of time does lessen PI, though it is possible that individual differences make an important contribution. For example, in the two-target condition of Experiment 1, almost two thirds of the sample did experience a decline in PI as the inter-trial interval was extended, with that reduction varying between 4 and 44%. Conversely, 23% of the sample showed a stronger PI effect at the longer interval (and 13% experienced no change). There was far less evidence for PI declining over the inter-trial interval in the four-target condition, as expected given the previous analysis, but in Experiment 2, almost half the sample did show some reduction in PI after the long inter-trial interval (the PI effect declining between 1 and 29%), whereas for 43%, PI became stronger. Understanding the cognitive profile of people who can and cannot use time to manage PI would be an important avenue for future research.

While the present data appear problematic for temporal models of forgetting, Experiment 3 found PI despite the secondary task presented within the inter-trial interval. This suggests that non-specific retroactive interference may also not be able to eliminate PI, and the combined evidence indicates a relatively durable form of PI. This durability has important implications for understanding the memory processes behind these effects. One possibility is that PI is produced by a lingering working memory of events on the prior trial (see Makovski and Jiang, 2008), but given the limitations with working memory, this seems unlikely. Instead, it is possible that long-term memory plays a role in PI. This interpretation is consistent with Oberauer et al. (2017), who proposed that PI results from a familiarity signal in long-term memory, and Shoval and Makovski (2021), who have argued that PI occurs during retrieval.

Some additional evidence for long-term memory involvement came from the repeated condition of Experiment 2, where the targets were drawn from a very small pool of stimuli. There was evidence for this manipulation playing at least a modest role in the task – the Bayesian analysis suggested a larger PI effect when targets were repeated throughout the study, rather than being unique. While this effect was just above the significance threshold, it is likely it would have been conventionally significant had the planned sample size been achieved.

Still, there are objections to this interpretation due to the stimuli employed here. In Experiments 1 and 3, and in the unique condition of Experiment 2, targets were artificial, unfamiliar images presented briefly and on a single occasion within the encoding array. Participants would therefore need to rapidly create new long-term memories of these stimuli. While this may seem unlikely, research into another form of unfamiliar and difficult to verbalize stimuli – patterns in auditory noise – has demonstrated its rapid integration into long-term memory (e.g., Agus et al., 2010; Viswanathan et al., 2016). It is also plausible that the “long-term” memory behind PI has a limited lifetime (e.g., tens of seconds to minutes, rather than hours or days). Endress and Potter (2014), for example, suggested that a large capacity, temporary form of memory contributes to PI, and the lifetime of this memory has been estimated to be a maximum of a few minutes. The memories underlying the PI in the present study may be of a similar nature. Indeed, in verbal memory, PI has been shown to dissipate over 45 s (Kincaid and Wickens, 1970) or 2-min (Loess and Waugh, 1967) delays.

A relatively long-lasting form of passive memory is also compatible with McKeown et al.’s (2014) active-passive model, in which old, residual memories are passively maintained over lengthy delays and are resistant to non-specific interference. However, there is a potential paradox emerging from this interpretation, as actively maintained visual stimuli are quickly forgotten over a retention interval, as already discussed (e.g., Ricker and Cowan, 2010, 2014; Pertzov et al., 2013; Mercer and Barker, 2020). It may be better, therefore, to view PI as produced by a durable yet not entirely accurate representation of old items. Evidence that PI can be produced by stimuli only resembling a prior target was reported by Mercer and Fisher (2022), but a broadly accurate representation of an old target may still be sufficient to produce PI. Conversely, in tasks requiring participants to remember an array of visual stimuli over a retention interval, an exact representation of that array is required to respond accurately. Further investigation of this issue would be beneficial, though as performance on Positive trials was far from perfect, despite the short retention intervals used, it highlights the difficulties of retaining an exact representation of a visual stimulus shown for a brief period.

Performance on the Positive trials also raises another issue, concerning the possibility of the probe interfering with encoding of the targets, especially at the short inter-trial interval used in Experiments 1 and 2. Accuracy was higher on Positive trials when the 8 s inter-trial interval was used, rather than 300 ms. The recovery in performance on Positive trials after the longer inter-trial interval could be due to reduced interference from the sensory memory of the probe, though it should be noted that even at the 300 ms inter-trial interval, the gap between the target and probe was 1.4 s, on average, when factoring in mean response time. This delay may therefore be beyond the traditional lifetime of (visual) sensory memory (< 1 s, e.g., Sligte et al., 2010). Nonetheless, the improvement on Positive trials over the inter-trial interval is interesting and could reflect the lingering contribution of working memory, a temporal distinctiveness effect, or possibly event segmentation, where events on Trial *N*-1 are more likely to be incorrectly merged with events on Trial *N* at a short inter-trial interval (see Ricker et al., 2014, for a discussion). If interference was produced by the probe – a potential factor in the recent-probes task – it would have been equivalent for the RN and NRN probes, so it does not undermine the PI effect examined here. Even so, these data show that there can still be other roles for time in visual working memory.

Further investigation of the role of temporal factors in visual working memory would be useful, and future research may also be able to address some limitations with the present study. Due to the coronavirus pandemic, all three experiments were conducted online. While these experiments were still able to find PI in line with laboratory-based studies, there is a loss of experimental control, particularly over the environment in which the study was completed. We were also unable to restrict participants from completing more than one experiment, so any participants who completed two or three experiments would have had additional familiarity with the task and procedure (though the PI effect, being relatively subtle, may be difficult to consciously control – especially on the response time measure). Additionally, given the need to reduce the length of the overall experiment, the maximum inter-trial interval tested was 8 s. While this created a relatively long duration over which an RN item could be lost from memory (the time passing since the RN probe was viewed as a target could be up to 16.1 s, depending on response time), it is possible that a clearer reduction in PI would be observed at much longer intervals (e.g., Loess and Waugh, 1967; Kincaid and Wickens, 1970). It would be useful to assess this in future work (though see McKeown et al., 2020, Experiment 3, for a demonstration of PI after a 30 s inter-trial interval).

In conclusion, despite some doubts about the role of PI in visual memory, the present study reported a form of item-specific PI when demand on working memory capacity was high (Experiment 1), when item-non-specific PI was prevalent (Experiment 2), and when retroactive interference from a secondary task was present (Experiment 3). PI appears to be relatively robust and capable of affecting performance under a variety of different circumstances, suggesting a passive visual memory endures alongside actively maintained visual information. This passive representation appears to possess characteristics more akin to long-term, rather than working, memory.

## Data Availability Statement

The datasets presented in this study can be found in online repositories. The names of the repository/repositories and accession number(s) can be found below: The datasets for this study can be found in the Open Science Framework project “Tracking Proactive Interference in Visual Memory: https://osf.io/a25dz/.”

## Ethics Statement

The studies involving human participants were reviewed and approved by the Faculty of Education, Health and Wellbeing Ethics Committee, University of Wolverhampton. The patients/participants provided their written informed consent to participate in this study.

## Author Contributions

TM designed and directed the project. TM, R-JJ, RL, and FW were equally involved in participant recruitment, data collection, and data analysis. TM wrote the manuscript in consultation with R-JJ, RL, and FW. All authors contributed to the article and approved the submitted version.

## Conflict of Interest

The authors declare that the research was conducted in the absence of any commercial or financial relationships that could be construed as a potential conflict of interest.

## Publisher’s Note

All claims expressed in this article are solely those of the authors and do not necessarily represent those of their affiliated organizations, or those of the publisher, the editors and the reviewers. Any product that may be evaluated in this article, or claim that may be made by its manufacturer, is not guaranteed or endorsed by the publisher.

## Footnotes

1
https://psych.hanover.edu/research/exponnet.html
